# Characteristics and transcriptomic analysis of scar tissues on the inner uterine cavity wall in patients with intrauterine adhesions

**DOI:** 10.3389/fphys.2022.990009

**Published:** 2022-12-22

**Authors:** Waixing Li, Pan Gu, Bingsi Gao, Lingxiao Zou, Aiqian Zhang, Huan Huang, Xingping Zhao, Dabao Xu, Chunxia Cheng

**Affiliations:** ^1^ Department of Obstetrics and Gynecology, The Third Xiangya Hospital of the Central South University, Changsha, Hunan, China; ^2^ The Obstetrics and Gynecology Hospital of Fudan University, Shanghai, China

**Keywords:** scar tissue, uterine smooth muscle, intrauterine adhesions, RNA-Seq, transcriptomic feature

## Abstract

**Introduction:** It has been previously reported that intrauterine adhesions (IUAs) are the main cause of uterine infertility. However, the histological origin of scar tissue present on the inner wall of the uterine cavity with IUAs has not been previously studied, which is particularly necessary for follow‐up research and prevention and treatment.

**Methods:** In this study, myometrium with normal uterus were assigned to the control group and scar tissues with IUAs were assigned to the experimental group. And pathological characteristics and transcriptomic were analyzed between the two groups.

**Results:** We founded no difference was noted in the histological morphology and the α-SMA expression between the experimental and control groups. A total of 698 differentially expressed genes were identified between the two groups. Gene Ontology (GO) analyses revealed that the DEGs were significantly enriched in cell proliferation, AP-1 complex formation, and angiogenesis. Kyoto Encyclopedia of Genes and Genomes (KEGG) pathway analyses revealed that the target genes were significantly enriched in the AGE-RAGE, FOXO and TNF signaling pathway.

**Discussion:** As far as we know, this is the first study to propose that the scar tissues are mainly derived from the myometrium and the first one to report differentially expressed genes in the scar tissues of IUAs.

## 1 Introduction

Intrauterine adhesions (IUAs) are primarily caused by the failure of regeneration and repair of functional layer, which results in the formation of fibrous scar tissue following endometrial basal layer injury (Zhang et al., 2019). It has been previously reported that IUAs are the main cause of uterine infertility, which is often secondary to multiple uterine cavity operations (Rein et al., 2011). There are two main points that need to be considered during the treatment of IUAs. These include repair of the morphology of uterine cavity and repair of uterine function (mainly to repair the coverage of the endometrium). Hysteroscopic surgery is the gold standard that is commonly used for restoring normal uterine morphology in patients with IUAs (Khan and Goldberg, 2018; Dreisler and Kjer, 2019). In the past few years, significant progress has been made in the field of hysteroscopic technology (Huang et al., 2018; Zhao et al., 2020; Zhu et al., 2020). Additionally, significant progress has been made in the research focused on addressing the issue of high recurrence rate after operation (Huang et al., 2020a; Huang et al., 2020b). However, no breakthrough achievement has been made in the repair of inner wall of the uterine cavity. Particularly, the local environment caused by scarring is not conducive to the growth of endometrium.

Certain studies believed that endometrial fibrosis is the main pathological change (Zhang et al., 2019), however, these studies ignored the pathological changes caused by the myometrial damage of the inner wall of the uterine cavity. Thus, these studies failed to explain the mechanism involved in the formation of a large number of scars on the inner wall of the uterine cavity in patients with severe IUAs. These scars are generally very harmful, and often lead to scar contracture of the uterine cavity, reduction of the volume of the uterine cavity, and lack of blood flow on the inner wall of the uterine cavity, mainly due to scar coverage. Further, this leads to the inability of the endometrium to grow well (Zhao et al., 2020). The histological origin of scar tissue present on the inner wall of the uterine cavity with IUAs has not been previously studied, which is particularly necessary for follow‐up research and prevention and treatment of scarring on the inner wall of the uterine cavity.

To date, most of the studies published on IUAs mainly focused on the evaluation of expression alterations for one or several specific genes. Additionally, the systematic studies on the differentially expressed genes or the primary pathways implicated in the initiation of scar tissue are seldom reported. Importantly, only a few studies have compared the expression profiles of miRNAs between endometrial tissues obtained from patients with IUAs and normal endometrial tissues using microarray technique (Li et al., 2016; Liu et al., 2016; Ning et al., 2018). Transcriptomic analysis can provide important insights into the disease pathogenesis that affect clinical management. In addition to this, previous IUAs treatment strategies primarily aimed to restore the shape of the uterine cavity and endometrial regeneration (Dreisler and Kjer, 2019; Lee et al., 2021). However, we found that the distance between two uterine horns with IUAs was shorter than the one reported in cases without IUAs. Therefore, it was speculated that the scar tissue with IUAs might primarily arise from muscle layer rather than endometrium. The contracture ring formed by the myometrium scar tissue could possibly cause gradual contracture of the uterus, which could explain the clinical phenomenon of shortening of the uterine angle distance in patients with IUAs. This hypothesis holds great significance in terms of research and treatment of IUAs, and indicates that endometrial therapy alone provides limited benefit.

The present study involved the collection of scar tissue from 16 patients with IUAs and normal myometrial tissue from 6 patients with normal uterus, for pathological analysis and transcriptomic expression comparison. This study particularly aimed to provide a reference for clinical treatment and a new perspective for studying the pathogenesis of IUAs.

## 2 Materials and methods

### 2.1 Participants

**FIGURE 1:**
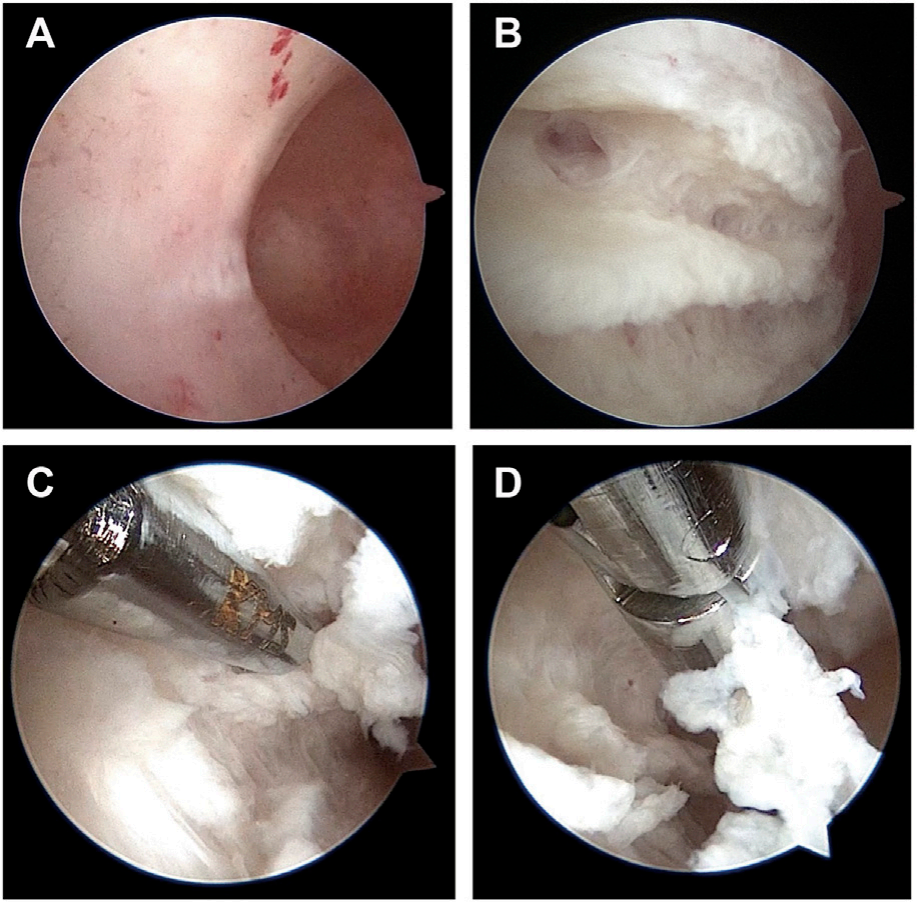
Scar tissue under hysteroscopy in patients with intrauterine adhesions. **(A)**. Scar tissue surface of inner wall of uterine cavity. **(B)**. Scar tissue image after cutting with scissors. **(C)**. Using scissors to cut scar tissue. **(D)**. Using forceps to clip scar tissue out of the uterine cavity.

In this study, 6 patients with a normal uterus were found eligible to be assigned to the control group and 16 patients with IUAs were assigned to the experimental group. The inclusion criteria for the experimental group were as follows: 1) patients with moderate or severe IUAs (The American Fertility Society, 1988); and 2) patients with scar tissues in the uterine cavity, scar tissues refer to the tissue surface on the inner uterine cavity without endometrial covering, without endometrial gland opening and blood flow, with white or grayish white color and hard, and dense tissue can be seen after cutting (Figure1). The inclusion criteria of the control group were as follows: 1) patients without endometrium and myometrium lesions, which were confirmed by MRI and pathological results; and 2) patients who underwent hysterectomy for cervical or ovarian lesions. The exclusion criteria were as follows: patients with diffuse uterine lesions, such as adenomyosis, adenomyoma, and endometrial cancer, which were confirmed by MRI and pathological results.

The tissues of 6 patients with IUAs and 6 subjects with normal uterus were subjected to pathological analysis. The mean age of the experimental group and the control group were 31.3 ± 4.0 and 36.8 ± 5.1, respectively, with no statistically significant difference existing between these two groups. The tissues of 10 patients with IUAs and 5 subjects with normal uterus were provided for sequencing analyses. And there was no significant difference in age, gravidity, parity, abortion, height, weight, and BMI between these two groups (*p* > .05) (Table1).

**TABLE 1:** Comparison of baseline characteristics between the control group and the experimental group for sequencing analyses.

Variable	The control group	The experimental group	*p*
Age (years)	35.8 ± 5.0	31.1 ± 5.9	.448
Gravidity	2.6 ± 2.5	2.8 ± 1.7	.560
Parity	1.6 ± .6	.7 ± 1.3	.390
Abortion	1.0 ± 2.2	2.1 ± .9	.074
Height (cm)	159.8 ± 4.5	160.1 ± 4.0	.581
Weight (kg)	56.4 ± 4.3	56.2 ± 9.8	.269
BMI	22.1 ± 1.2	21.9 ± 3.2	.074

### 2.2 Tissue sampling and treatment

Scar tissues sampling process (Figure1): the sterile scissors were used to remove scar tissues and the tissues were putted on sterile gauze during the hysteroscopic surgery, then the tissues were preserved in the RNA-later solution and transferred to the −80°C or were fixed in 4% paraformaldehyde (PFA) solution at 4°C for subsequent sequencing analyses or pathological section analyses respectively; myometrium sampling process: the uterus were putted in a sterile disk after hysterectomy, and were dissected by a sterile blade with scraping off the endometrium, then the superficial myometrium tissues were taked by sterile scissors and were transferred to the −80°C or were fixed in 4% PFA solution at 4°C for subsequent sequencing analyses or pathological section analyses respectively.

### 2.3 Histologic examination with hematoxylin and eosin (H&E) staining

The HE staining procedure was conducted as previously described (Feng et al., 2020). The tissue samples were routinely subjected to alcohol gradient dehydration, xylene decoloring, and paraffin embedding. Then, the samples were sectioned, stained with hematoxylin-eosin (HE), and sealed with neutral gum.

### 2.4 Detection of tissue fibrosis with Masson staining

The Masson staining procedure was conducted as described previously (Feng et al., 2020). To determine the degree of collagen fiber accumulation, 18 ×400 visual fields from 6 individual sections were randomly selected for calculating the ratios of the areas of Van Gieson-stained interstitial fibrosis to the total field area using the ImageJ software.

### 2.5 Immunohistochemistry (IHC)

The immunohistochemistry procedure was conducted as described elsewhere (Feng et al., 2020). The tissue sections were deparaffinized, treated with 3% H2O2 for 25 min to inactivate endogenous peroxidases, heated in 10-mM citrate buffer at 121°C for 30 min for antigen retrieval, blocked in 5% normal serum for 20 min, and incubated with a primary polyclonal rabbit anti-rat antibody specific for *α*-SMA (1:200 in PBS, Servicebio; GB111364) overnight at 4°C. After three extensive washes with PBS, the sections were incubated with a biotin-conjugated secondary antibody (1:2,000 in PBS, Servicebio; GB1213) for 60 min at 37°C. After further incubation with horseradish peroxidase (HRP)-labeled streptavidin, antibody binding was visualized with diaminobenzidine (DAB), and the sections were counterstained with hematoxylin for 30 s at room temperature based on the manufacturer’s instruction. Eighteen ×400 visual fields in six individual sections were randomly selected for calculating the integrated optical density (IOD) using the ImageJ software.

### 2.6 RNA extraction and sequencing

The total RNA of the tissue samples was extracted by using the Trizol method. The Nanodrop2000 spectrophotometer was used to detect the concentration and purity of RNA; agarose gel electrophoresis was performed to evaluate the integrity of the RNA, and the Agilent 2100 Bioanalyzer was employed to determine the RNA Integrity Number (RIN) value.

### 2.7 Quality control and RNA sequence data analysis

The sequencing data was filtered with the SOAPnuke (v1.5.2) by 1) removing reads containing sequencing adapter; 2) removing reads whose low-quality base ratio (base quality ≤ 5) was >20%; 3) removing reads whose unknown base (“N” base) ratio was >5%, afterwards clean reads were obtained and stored in the FASTQ format. The clean reads were mapped to the reference genome using the HISAT2 (v2.0.4). Bowtie2 (v2.2.5) was applied to align the clean reads to the reference coding gene set and the expression level of gene was calculated by RSEM (v1.2.12). The heatmap was drawn by pheatmap (v1.0.8) according to the gene expression in different samples. Essentially, differential expression analysis was performed using the DESeq2 (v1.4.5) with Q value ≤ 0.05. To acquire an insight into the change of phenotype, GO and KEGG enrichment analyses of annotated differentially expressed gene was performed by Phyper based on the hypergeometric test. The significant levels of terms and pathways were corrected by Q value with a rigorous threshold (Q value ≤ 0.05) by the Bonferroni method.

### 2.8 Statistical analysis

SPSS 23 (IBM Company, Chicago, IL) statistical analysis software was used for data analysis. The data were depicted using mean ± standard deviation. The Scheffe’s test or Fisher’s exact test were utilized for univariate analysis. All DEGs were input into the IPA software to identify statistically significantly difference (calculated by Fisher’s exact test) and biologically significantly modulated (using an algorithm to predict the direction of change) biological functions and pathways between the two groups. The test level was set at *α* = .05, and *p* < .05 was considered to indicate statistical significance.

## 3 Results

### 3.1 Pathological and immunohistochemical features

**FIGURE 2:**
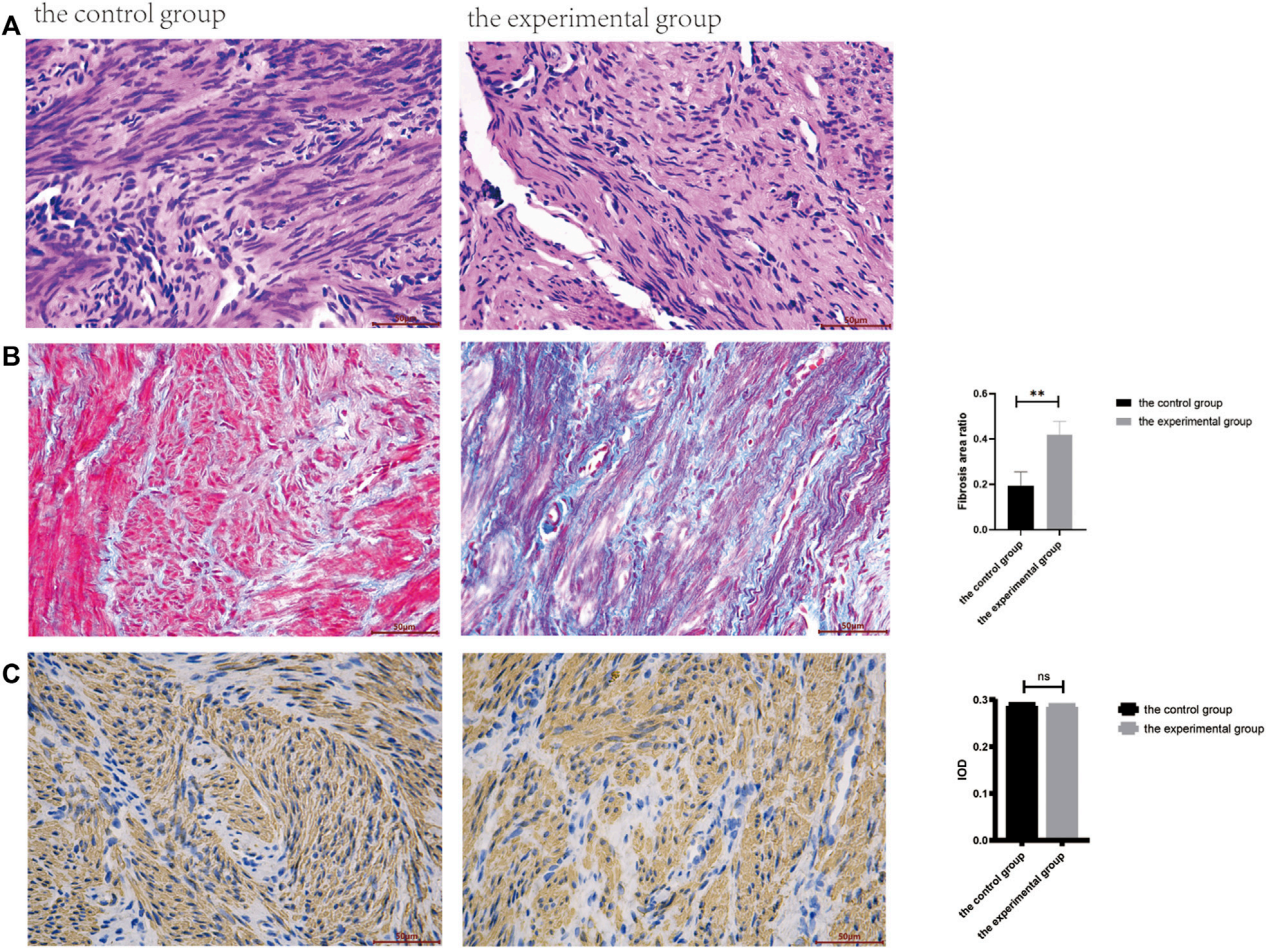
Pathological and immunohistochemical results between the control and the experimental groups. **(A)**. The HE staining (400ⅹ). **(B)**. The Masson staining (400ⅹ), the fibrosis area was calculated by ImageJ software, and there was significant statistical difference between the two groups (**p < .01). **(C)**. Immunohistochemical staining of smooth muscle specific marker *α*-SMA was compared between the two groups.

The present study included a total of 12 patients for pathological and immunohistochemical analyses. In particular, 6 patients with normal uterus were included in control group, and 6 patients with IUAs were included in experimental group. The morphological characteristics for these two groups of tissues were assessed by HE staining. As shown in Figure 1A, the uterine smooth muscle tissue in control group was characterized by the presence of neatly arranged smooth muscle cells, with tight intercellular structure. The scar tissue in experimental group exhibited sparely arranged smooth muscle fibers, with connective tissue hyperplasia in the intercellular stroma. In experimental group, very few endometrium residues were observed in case of two tissue samples, while the rest of the tissues showed morphological characteristics of smooth muscle tissue (Figure 2).

As shown in Figure 1B, the blue staining corresponded to collagen fibers. The results for Masson staining showed that the area of fibrosis in experimental group was more as compared to control group (*p* < .05).

As shown in Figure 1C, the presence of α‐SMA as smooth muscle tissue marker was determined by immunohistochemistry, wherein presence of brown nuclei indicated positive staining. No statistically significant differences were recorded between these two groups (*p* > .05).

### 3.2 Evaluation of sequencing quality and gene expression levels

**FIGURE 3:**
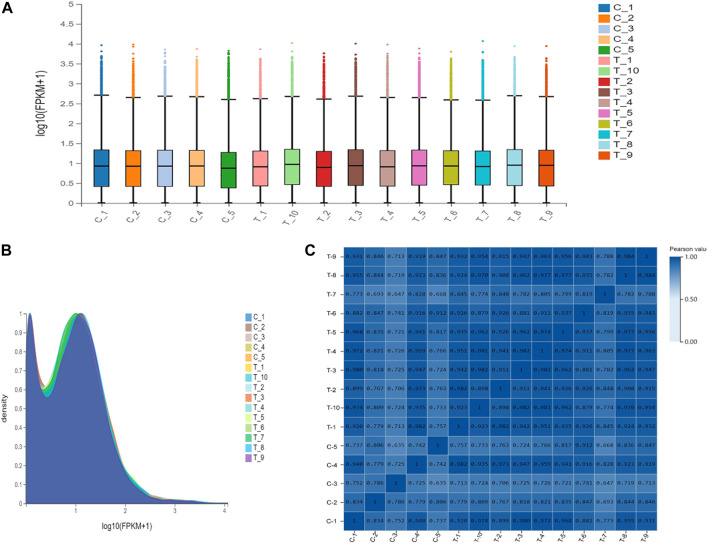
Gene expression levels in the control group and the experimental group. **(A)**. Express the boxplot. The *X*-axis is the sample name, and the *Y*-axis is log10(FPKM+1). The boxplot of each region corresponds to five statistics (upper limit, upper quartile, median, lower quartile, and lower limit from top to bottom, where the upper limit and lower limit do not consider outliers). **(B)**. Gene density map. The *X*-axis is log10(FPKM+1), and the *Y*-axis is the gene density, that is, the ratio of the number of genes under this expression level to the total number of expressed genes. **(C)**. Sample correlation heat map. The *X* and *Y* axes represent each sample. The color represents the correlation coefficient (the darker the color, the higher the correlation).

For transcriptomics analysis, a total 15 patients were included, wherein 5 patients with normal uterus belonged to control group, while 10 patients with IUAs were included in experimental group. RNA sequencing was performed for the two groups. After mass filtration and ribosomal RNA exclusion, an average of 4.8 GB data was obtained per sample. The filtered data were used for gene expression analysis. It was found that overall gene expression was similar for the two groups of tissues (Figure 3).

### 3.3 Transcriptomic features

**FIGURE 4:**
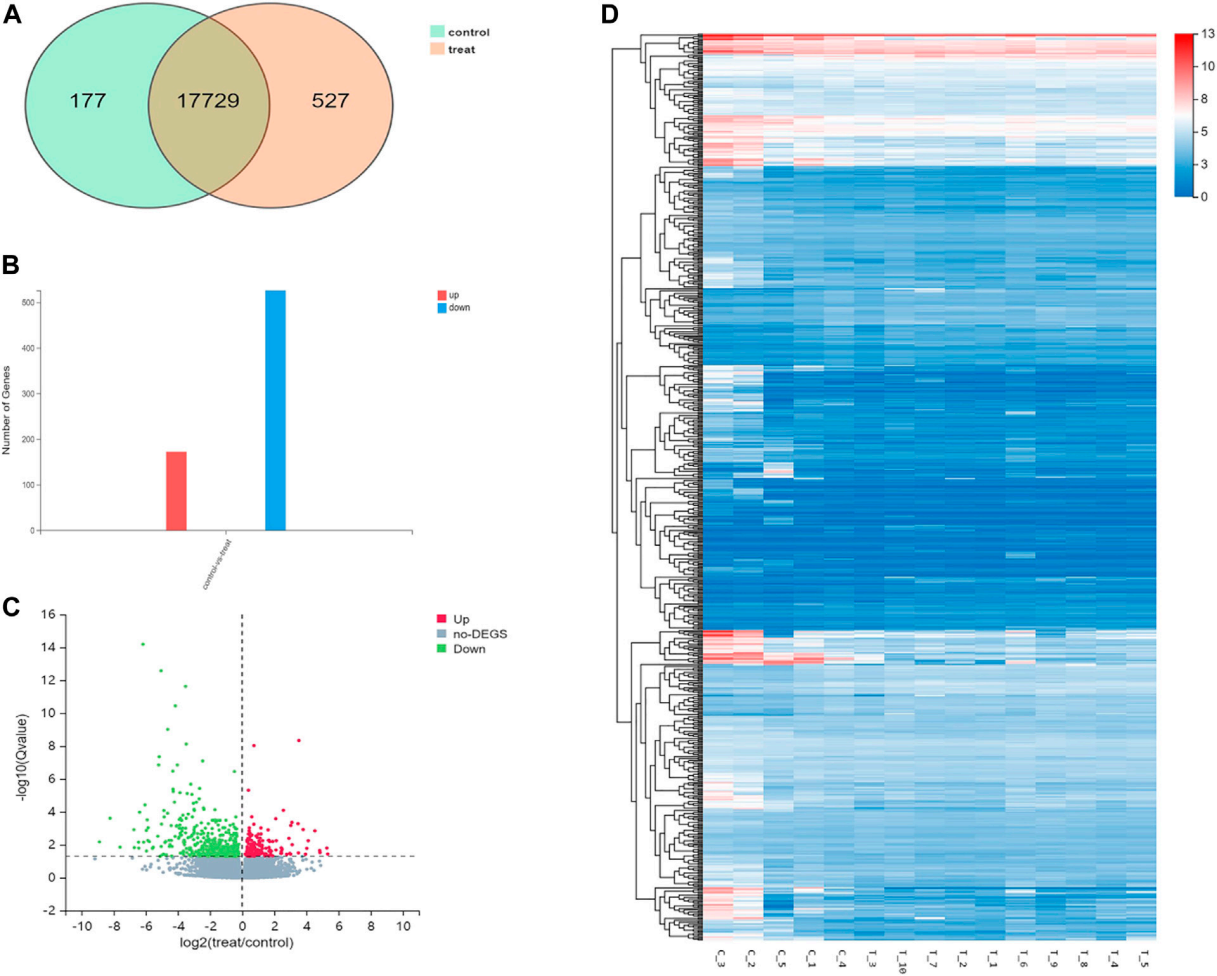
Differential gene expression in the control group and the experimental group. **(A)**. The intersecting genes in two groups. **(B)**. Number of differential genes. **(C)**. Differential gene volcano map. **(D)**. Hierarchical clustering analysis of genes with differential expression. On the horizontal axis is the log2 of the sample (expression value + 1), and on the vertical axis is the gene. The redder the color block, the higher the expression, the bluer the color, the lower the expression.

**FIGURE 5:**
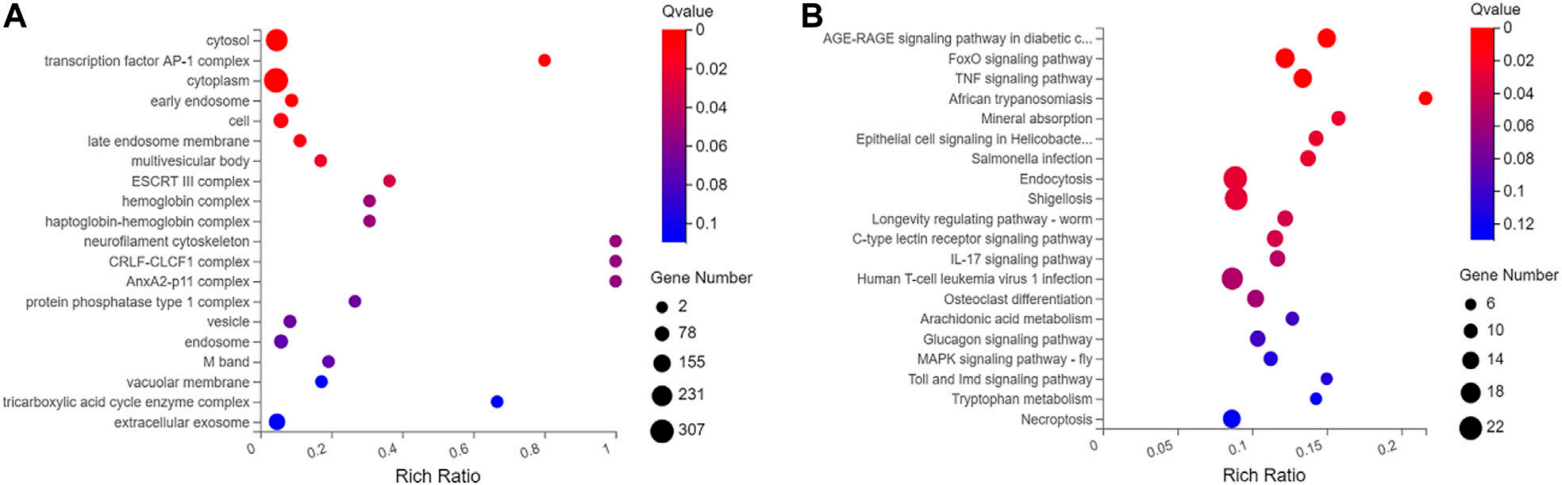
Gene ontology and Kyoto Encyclopedia of Genes and Genomes analysis of 698 differential genes. **(A)** GO function enrichment analyses of 698 differentially expressed genes between scar tissue with IUAs and myometrium with normal uterus. **(B)** KEGG pathway analysis of 698 differentially expressed genes between scar tissue with IUAs and myometrium with normal uterus.

When compared with control group, 698 differentially expressed genes were recorded in experimental group (*p* < .05), which included 527 downregulated genes and 171 up‐regulated genes. Gene Ontology (GO) analyses showed that DEGs were significantly enriched in cell proliferation, AP-1 complex formation, and angiogenesis. Kyoto Encyclopedia of Genes and Genomes (KEGG) pathway analyses showed that the target genes were significantly enriched in AGE-RAGE, FOXO, and TNF signaling pathway (Figures 4, 5).

### 3.4 Gene selection and prediction

**FIGURE 6:**
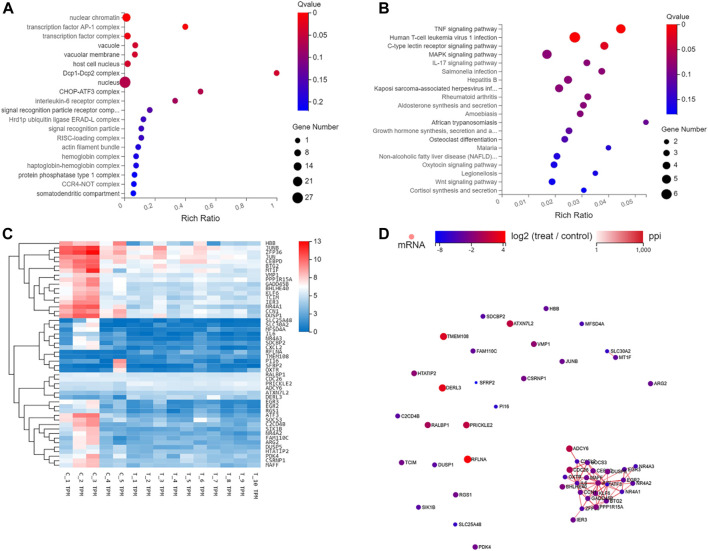
GO, KEGG, Hierarchical clustering, and gene regulatory network analysis of the 50 genes with the most significant differences. **(A)** GO function enrichment analyses of 50 differentially expressed genes between scar tissue with IUAs and myometrium with normal uterus. **(B)** KEGG pathway analysis of the most 50 differentially expressed genes between scar tissue with IUAs and myometrium with normal uterus. **(C)** Hierarchical clustering analysis of genes with differential expression. **(D)** Differentially expressed miRNA-differentially expressed gene regulatory network. The red and green colors denote upregulation and downregulation, respectively.

On the bases of degree values, ATF3, NR4A1, CCN1, DUSP1, zfp36, rflna, Gadd45b, atxn7l2, cxcl2, and bhlhe40 were identified as the top 10 hub genes. Further analysis of mRNA molecular interaction network, generated using 50 genes that exhibited most significant differences, showed that the target genes were enriched in TNF, immune T cells and MAPK signaling pathway. Importantly, the JUN, with largest number of nodes, was found to be the central node of the traffic network. GO analysis showed that DEGs were significantly enriched in cell proliferation, AP-1 complex and transforming factor complex (Figure 6; Tables 2, 3).

**TABLE 2:** GO function enrichment analyses of the top 50 differentially expressed miRNAs.

Gene	Q value	GO function	Treat/control
“ATF3”	6.47E-15	GO:0000790///nuclear chromatin	Down
“NR4A1”	2.62E-13	GO:0000790///nuclear chromatin	Down
“CCN1”	2.35E-12	GO:0062023///collagen-containing extracellular matrix	Down
“DUSP1”	3.55E-11	GO:0005634///nucleus	Down
“ZFP36”	9.56E-10	GO:0000178///exosome (RNase complex)	Down
“RFLNA”	4.54E-09	GO:0005856///cytoskeleton	Up
“GADD45B”	7.47E-09	GO:0005634///nucleus	Down
“ATXN7L2”	9.28E-09	NA	Up
“CXCL2”	4.42E-08	GO:0005576///extracellular region	Down
“BHLHE40”	7.92E-08	GO:0000790///nuclear chromatin	Down
“NR4A3”	1.39E-07	GO:0000790///nuclear chromatin	Down
“NR4A2”	1.40E-07	GO:0000790///nuclear chromatin	Down
“SIK1B”	3.31E-07	GO:0005634///nucleus	Down
“RALBP1”	3.49E-07	GO:0005829///cytosol	Down
“IER3”	2.04E-06	GO:0005575///cellular_component	Down
“KLF6”	3.70E-06	GO:0001650///fibrillar center	Down
“EGR3”	4.08E-06	GO:0000790///nuclear chromatin	Down
“ADCY6”	4.77E-06	GO:0005886///plasma membrane	Up
“SDCBP2”	5.73E-06	GO:0005634///nucleus	Down
“EGR2”	6.80E-06	GO:0000790///nuclear chromatin	Down
“BTG2”	7.53E-06	GO:0005634///nucleus	Down
“MAFF”	8.57E-06	GO:0000790///nuclear chromatin	Down
“DUSP5”	1.60E-05	GO:0005634///nucleus	Down
“CSRNP1”	2.55E-05	GO:0016021///integral component of membrane	Down
“C2CD4B”	2.55E-05	GO:0005634///nucleus	Down
“RGS1”	2.95E-05	GO:0005737///cytoplasm	Down
“SLC30A2”	3.69E-05	GO:0005774///vacuolar membrane	Down
“PPP1R15A”	5.63E-05	GO:0000164///protein phosphatase type 1	Down
“TCIM”	6.91E-05	GO:0005634///nucleus	Down
“JUN”	6.91E-05	GO:0035976///transcription factor AP-1 complex	Down
“HTATIP2”	7.29E-05	GO:0005829///cytosol	Down
“DERL3”	7.98E-05	GO:0005785///signal recognition particle receptor complex	Down
“CEBPD”	8.19E-05	GO:0000790///nuclear chromatin	Down
“MFSD4A”	8.23E-05	GO:0016020///membrane	Down
“PI16”	1.05E-04	GO:0005576///extracellular region	Down
“IL6”	1.20E-04	GO:0005576///extracellular region	Down
“JUNB”	1.35E-04	GO:0035976///transcription factor AP-1 complex	Down
“FAM110C”	1.57E-04	GO:0005634///nucleus	Down
“PRICKLE2”	1.96E-04	GO:0005634///nucleus	Up
“PDK4”	1.99E-04	GO:0005739///mitochondrion	Down
“OXTR”	1.99E-04	GO:0005623///cell	Down
“CDC26”	1.99E-04	GO:0005680///anaphase-promoting complex	Down
“ARG2”	2.41E-04	GO:0005759///mitochondrial matrix	Down
“SFRP2”'	2.41E-04	GO:0062023///collagen-containing extracellular matrix	Down
“SOCS3”	2.41E-04	GO:0005942///phosphatidylinositol 3-kinase complex	Down
“TMEM108”	2.57E-04	GO:0005575///cellular_component	Up
“MT1F”	2.88E-04	GO:0048471///perinuclear region of cytoplasm	Down
“HBB”	3.01E-04	GO:0005576///extracellular region GO:0000407///phagophore assembly site	Down
“VMP1”	3.02E-04	GO:0005739///mitochondrion	Down
“SLC25A48”	3.02E-04		Down

**TABLE 3:** KEGG pathway analysis of the top 50 differentially expressed miRNAs.

Gene	Q value	KEGG signaling pathway
“ATF3”	6.47E-15	NA
“NR4A1”	2.62E-13	MAPK, PI3K/Akt signaling pathway, et al.
“CCN1”	2.35E-12	NA
“DUSP1”	3.55E-11	MAPK signaling pathway
“ZFP36”	9.56E-10	Human T-cell leukemia virus 1 infection
“RFLNA”	4.54E-09	NA
“GADD45B”	7.47E-09	MAPK, FoxO, p53 signaling pathway, Apoptosis, et al.
“ATXN7L2”	9.28E-09	NA
“CXCL2”	4.42E-08	Cytokine-cytokine receptor interaction, NF-kappa B, TNF signaling pathway
“BHLHE40”	7.92E-08	Circadian rhythm
“NR4A3”	1.39E-07	Transcriptional misregulation in cancer
“NR4A2”	1.40E-07	Aldosterone synthesis and secretion
“SIK1B”	3.31E-07	Glucagon signaling pathway
“RALBP1”	3.49E-07	Ras signaling pathway
“IER3”	2.04E-06	NA
“KLF6”	3.70E-06	NA
“EGR3”	4.08E-06	C-type lectin receptor signaling pathway
“ADCY6”	4.77E-06	Purine metabolism, Endocrine resistance, et al.
“SDCBP2”	5.73E-06	NA
“EGR2”	6.80E-06	C-type lectin receptor signaling pathway, Human T-cell leukemia virus 1 infection
“BTG2”	7.53E-06	RNA degradation
“MAFF”	8.57E-06	NA
“DUSP5”	1.60E-05	MAPK signaling pathway
“CSRNP1”	2.55E-05	NA
“C2CD4B”	2.55E-05	NA
“RGS1”	2.95E-05	NA
“SLC30A2”	3.69E-05	NA
“PPP1R15A”	5.63E-05	Protein processing in endoplasmic reticulum
“TCIM”	6.91E-05	NA
“JUN”	6.91E-05	Endocrine resistance, Apoptosis, MAPK, ErbB and Wnt signaling pathway, et al.
“HTATIP2”	7.29E-05	NA
“DERL3”	7.98E-05	Protein processing in endoplasmic reticulum
“CEBPD”	8.19E-05	NA
“MFSD4A”	8.23E-05	NA
“PI16”	1.05E-04	NA
“IL6”	1.20E-04	EGFR tyrosine kinase inhibitor resistance, PI3K-Akt signaling pathway, et al.
“JUNB”	1.35E-04	Osteoclast differentiation, TNF signaling pathway, et al.
“FAM110C”	1.57E-04	NA
“PRICKLE2”'	1.96E-04	Wnt signaling pathway
“PDK4”	1.99E-04	NA
“OXTR”	1.99E-04	Calcium signaling pathway, cAMP signaling pathway, et al.
“CDC26”	1.99E-04	Human T-cell leukemia virus 1 infection, Cell cycle, et al.
“ARG2”	2.41E-04	Arginine biosynthesis, Arginine and proline metabolism et al.
“SFRP2”	2.41E-04	Wnt signaling pathway
“SOCS3”	2.41E-04	Jak-STAT,TNF, Insulin signaling pathway, et al.
“TMEM108”	2.57E-04	NA
“MT1F”	2.88E-04	Longevity regulating pathway—worm, Mineral absorption
“HBB”	3.01E-04	African trypanosomiasis, Malaria
“VMP1”	3.02E-04	Autophagy—animal
“SLC25A48”	3.02E-04	NA

## 4 Discussion

In order to identify more effective treatment strategies for IUAs, it is important to clarify the underlying mechanisms responsible for the occurrence and development of IUAs. Many studies have previously summarized the pathological changes associated with IUAs (Taskin et al., 2000; Xu et al., 2017; Khan and Goldberg, 2018; Leung et al., 2021). These included endometrial fibrosis, endometrial scarring, and loss or thinning of endometrium (Cheng et al., 2020). Endometrial fibrosis is the main pathological feature of IUAs (Rockey et al., 2015). In fact, endometrial regeneration has been previously shown to be a key factor that affects the prognosis of IUAs. However, these studies ignored the pathological changes caused by myometrial injury in the inner wall of the uterine cavity. Scarring of the inner wall of the uterine cavity is one of the important factors that hinder the regeneration and repair of the endometrium. At present, no study is available on the mechanism involved in scar tissue formation and source of scar tissue, which forms the basis of follow‐up research and prevention of scarring of the inner wall of the uterine cavity. A study conducted in our laboratory reported that the angular distance in patients with IUAs was shorter than that of normal women (Lei et al., 2022), which could not be explained by the mechanism of endometrial fibrosis. In order to explain these phenomena and further explore the mechanism involved in the occurrence and development of IUAs, a control study was designed, wherein scar tissue was collected from patients with IUAs and the myometrial tissue was obtained from normal uterine patients, for pathological section comparison. Interestingly, when compared with normal myometrial tissue, scar tissue showed the histological characteristics of smooth muscle cells. Additionally, the fibrotic area was higher than that of normal myometrial tissue. Importantly, no differences were recorded in the expression of α‐SMA for the two groups. α‐SMA is the specific marker of smooth muscle cells. Therefore, it was speculated that the scar tissue for IUAs might primarily arise from muscle layer rather than endometrium. In addition to endometrial damage, myometrial tissue on the surface of the uterine cavity was also found to be damaged and fibrosis was observed. These observations could possibly explain the occurrence of shortened uterine angular distance in patients with moderate and severe IUAs. Importantly, this angular distance would gradually shorten with the extension of the course of the disease, primarily owing to the formation of contracture ring by the myometrium scar tissue that could cause gradual contracture of the uterus. More importantly, if the problem of myometrial fibrosis really exists, it should be given importance equivalent to endometrial fibrosis, because myometrial fibrosis can hardly recover once the uterine cavity becomes smaller. At the same time, the coverage of scars contributes to difficulties in endometrial regeneration, which directly affects the prognosis of pregnancy.

In order to further verify this conjecture, scar tissues were collected from 10 patients with IUAs, while normal myometrial tissue was obtained from 5 patients with normal uterus, for transcriptomic sequencing analysis. The results showed that the majority of the genes exhibited similar expression levels in the tissues obtained from the two groups, indicating that there were no differences between the two sources of tissues. These results verified the hypothesis that most of the scar tissue came from the myometrium. As far as we know, the present study is first to investigate and compare the transcriptomic characteristics between uterine scar tissue and matched control uterine smooth muscle tissue. This finding holds great significance for clinical treatment, and it also provides a new idea for assessing the pathogenesis of IUAs. Thus, the patients with IUAs need to be treated as soon as diagnosis is made. Importantly, for patients with IUAs with fertility requirements, it is necessary to inform them regarding the necessity to plan pregnancy at the earliest after the completion of the treatment, particularly to avoid the gradual shrinkage of the uterus and affect the prognosis of pregnancy (Gao et al., 2019). The clinical treatment of IUAs no longer focuses on the promotion of endometrial regeneration alone. The treatment of scar tissue is also not restricted to complete resection. In fact, it is important to plough the scar tissue instead of cutting it off. When the scar tissue is cut off, the contracture ring can be cut off to alleviate the shrinkage process of the uterus. When the scar tissue is removed, it not only damages the potential intima, but it also forms a new myometrial contracture ring, which fails to achieve the ideal effect. Moreover, scar tissue can expose the healthy muscle layer that is rich in blood vessels, providing growth space and nutrition for the regenerated intima, after ploughing. In addition to this, cutting scar tissue also requires certain skills. In particular, cutting too deep would damage the muscle layer and easily cause bleeding, whereas cutting too shallow cannot achieve the therapeutic effect. Hysteroscopy of scar tissue and muscularis showed that the cut surface of scar tissue was dense and pale, with almost no blood vessels, while the cut surface of normal muscle layer is pink mesh and rich in blood vessels. Once the cut reaches the pink muscle layer, it indicates the depth is enough. Our research group has proposed the method of ploughing scar tissue by ploughing field method, during the early stage. Interestingly, it has been previously reported that the pregnancy outcome of this method is better as compared to traditional electrosurgical resection. These results confirmed this theory, and proved that this method is indeed safe and effective (Zhao et al., 2020).

At present, the studies available on the pathogenesis of mRNA network in scar tissue of IUAs are quite limited. In the past, a few studies collected endometrial tissue from the patients with IUAs and normal human endometrial tissue for transcriptome analysis. It was reported that the differentially expressed genes were related to cell adhesion process, negative regulation of growth, angiogenesis, cell connection assembly, negative regulation of cell migration, and Wnt signaling pathway. It was also observed that the target gene was related to multiple signal transduction pathways, such as Ras, Hippo, and MAPK (Jiang et al., 2019; Zong et al., 2021). In this study, some abnormally expressed genes were reported in scar tissue, suggesting that abnormal gene expression in uterine smooth muscle tissue might lead to the formation of scar tissue. Importantly, 678 genes exhibited significant differential gene expression between the scar tissue and normal myometrial tissue. GO analysis revealed that differential genes were enriched in cell proliferation, AP-1 complex and transforming factor complex, which were involved in the occurrence and development of scar tissue. IUAs were found to be closely related to the obstacle of tissue regeneration and repair and formation of fibrous scar tissue. Additionally, cell proliferation and angiogenesis are very important for promoting tissue regeneration and inhibiting fibrous scar. Previous studies have also shown that pathological mechanism for scar thin endometrium involved blocking of angiogenesis (Jiang et al., 2019). In addition, KEGG analysis suggested that the target genes enriched in AGE-RAGE, FOXO, and TNF signaling pathways. The degree of fibrosis is an important indicator of scar tissue. It was hypothesized that different pathways are involved in the development of scar tissue. Estrogen is known to promote the recovery of uterine morphology and reduce the probability of uterine fibrosis in the animal model of IUAs, which was mediated by inhibition of expression of TGF‐β and TNF‐α (Jiang et al., 2019). It has also been previously reported that endometrium with IUAs enriched in FOXO signaling pathway, as assessed by transcriptomic analysis (Yao et al., 2020). Another study reported that the AGE‐RAGE signaling pathway is also an important mechanism that is involved in fibrosis diseases of other organs (Sharma et al., 2021).

The analysis of molecular interaction network showed that “TNF, immune T cells and MAPK signaling pathway” was the most abundant network. Importantly, JUN was found to be the central node of the traffic network. It was characterized by the presence of largest number of nodes. These results were consistent with the findings of the previous studies (Lappas, 2018; Liu et al., 2020; Queckbörner et al., 2020). Jun (Choi et al., 2021), also known as AP‐1, is a transcription factor subunit. It is the transformation gene of avian sarcoma virus 17, which (Choi et al., 2021) has been previously shown to be involved in various cellular events, including cell proliferation, differentiation, survival, metabolism, hypoxia, angiogenesis, steroidogenesis, and prostaglandin (PG) production. GO analysis also showed that the differential genes were mainly related to cell proliferation, AP-1 complex and transforming factor complex. In addition to this, the 10 genes with the most significant differences have been previously reported to be related to apoptosis, cell proliferation, vascular remodeling, inflammation and immunity, damage repair, fibrosis, cell migration, and differentiation (Gay et al., 2011; Palumbo-Zerr et al., 2015; Cook et al., 2020; Cao et al., 2021; Ding et al., 2021; Liu et al., 2021; Olaloye et al., 2021; Radyk et al., 2021; Tang et al., 2021). These results were consistent with the findings of the previous studies conducted, which explored the occurrence and development mechanism of IUAs. However, there are no reports on the role of these genes in IUAs. Therefore, it is necessary to further explore the molecular mechanism of scar tissue, and provide a new perspective for the study of potential new biomarkers and therapeutic targets in the future.

## 5 Conclusion

This study was associated with certain limitations. In particular, the mechanism involved in the process was not verified in this study. Additionally, the study did not include endometrial tissue as control. Thus, the future studies should involve improved experiments, and results should be verified. Altogether, as far as we know, the present study is first to reveal that the scar tissue of IUAs mainly arise from the myometrium. The study also revealed the key pathways and networks involved in scar tissue, which would assist in further exploration and treatment of scarring of intrauterine wall of IUAs in the future.

## Data Availability

The sequencing data was deposited at NCBI under BioProject: PRJNA870310, available from https://www.ncbi.nlm.nih.gov/bioproject/PRJNA870310.
